# Tumour risks and genotype–phenotype correlations associated with germline variants in succinate dehydrogenase subunit genes *SDHB*, *SDHC* and *SDHD*

**DOI:** 10.1136/jmedgenet-2017-105127

**Published:** 2018-01-31

**Authors:** Katrina A Andrews, David B Ascher, Douglas Eduardo Valente Pires, Daniel R Barnes, Lindsey Vialard, Ruth T Casey, Nicola Bradshaw, Julian Adlard, Simon Aylwin, Paul Brennan, Carole Brewer, Trevor Cole, Jackie A Cook, Rosemarie Davidson, Alan Donaldson, Alan Fryer, Lynn Greenhalgh, Shirley V hodgson, Richard Irving, Fiona Lalloo, Michelle McConachie, Vivienne P M McConnell, Patrick J Morrison, Victoria Murday, Soo-Mi Park, Helen L Simpson, Katie Snape, Susan Stewart, Susan E Tomkins, Yvonne Wallis, Louise Izatt, David Goudie, Robert S Lindsay, Colin G Perry, Emma R Woodward, Antonis C Antoniou, Eamonn R Maher

**Affiliations:** 1 Department of Medical Genetics, University of Cambridge and NIHR Cambridge Biomedical Research Centre and Cancer Research UK Cambridge Cancer Centre and Cambridge University Hospitals NHS Foundation Trust, Cambridge, UK; 2 Department of Biochemistry, University of Cambridge, Cambridge, UK; 3 Department of Biochemistry and Molecular Biology, Bio21 Institute, University of Melbourne, Melbourne, Victoria, Australia; 4 Instituto René Rachou, Fundação Oswaldo Cruz, Belo Horizonte, Brazil; 5 Department of Public Health and Primary Care, University of Cambridge, Cambridge, UK; 6 West Midlands Regional Genetics service, Birmingham Women’s Hospital, Birmingham, UK; 7 Department of Clinical Genetics, Queen Elizabeth University Hospital, Glasgow, UK; 8 Yorkshire Regional Genetics Service, St. James’s University Hospital, Leeds, UK; 9 Department of Endocrinology, King’s College Hospital, London, UK; 10 Northern Genetics Service, Newcastle upon Tyne Hospitals NHS Foundation Trust, Newcastle upon Tyne, UK; 11 Peninsula Clinical Genetics Service, Royal Devon & Exeter Hospital, Exeter, UK; 12 Department of Clinical Genetics, Sheffield Children’s Hospital, Sheffield, UK; 13 Department of Clinical Genetics, St Michael’s Hospital, Bristol, UK; 14 Department of Clinical Genetics, Liverpool Women’s NHS Foundation Trust, Liverpool, UK; 15 Department of Medical Genetics, St. George’s University of London, London, UK; 16 Queen Elizabeth Medical Centre, Queen Elizabeth Hospital, Birmingham, UK; 17 Manchester Centre for Genomic Medicine, St Mary’s Hospital, Central Manchester University Hospitals NHS Foundation Trust, Manchester, UK; 18 East of Scotland Regional Genetics Service, Ninewells Hospital and Medical School, Dundee, UK; 19 Northern Ireland Regional Genetics Service, Belfast City Hospital, Belfast Health & Social Care Trust, Belfast, UK; 20 Department of Clinical Genetics, Addenbrooke’s Treatment Centre, Cambridge University Hospitals NHS Foundation Trust, Cambridge, UK; 21 The Wolfson Diabetes and Endocrine Clinic, Institute of Metabolic Science, Cambridge University Hospitals NHS Foundation Trust, Cambridge, UK; 22 Department of Clinical Genetics, Guy’s Hospital, London, UK; 23 Institute of Cardiovascular & Medical Sciences, University of Glasgow, Glasgow, Scotland

**Keywords:** cancer: endocrine, genetics, molecular genetics, oncology, genetic epidemiology

## Abstract

**Background:**

Germline pathogenic variants in *SDHB/SDHC*/*SDHD* are the most frequent causes of inherited phaeochromocytomas/paragangliomas. Insufficient information regarding penetrance and phenotypic variability hinders optimum management of mutation carriers. We estimate penetrance for symptomatic tumours and elucidate genotype–phenotype correlations in a large cohort of *SDHB/SDHC*/*SDHD* mutation carriers.

**Methods:**

A retrospective survey of 1832 individuals referred for genetic testing due to a personal or family history of phaeochromocytoma/paraganglioma. 876 patients (401 previously reported) had a germline mutation in *SDHB/SDHC*/*SDHD* (n=673/43/160). Tumour risks were correlated with in silico structural prediction analyses.

**Results:**

Tumour risks analysis provided novel penetrance estimates and genotype–phenotype correlations. In addition to tumour type susceptibility differences for individual genes, we confirmed that the *SDHD:*p.Pro81Leu mutation has a distinct phenotype and identified increased age-related tumour risks with highly destabilising *SDHB* missense mutations. By Kaplan-Meier analysis, the penetrance (cumulative risk of clinically apparent tumours) in *SDHB* and (paternally inherited) *SDHD* mutation-positive non-probands (n=371/67 with detailed clinical information) by age 60 years was 21.8% (95% CI 15.2% to 27.9%) and 43.2% (95% CI 25.4% to 56.7%), respectively. Risk of malignant disease at age 60 years in non-proband *SDHB* mutation carriers was 4.2%(95% CI 1.1% to 7.2%). With retrospective cohort analysis to adjust for ascertainment, cumulative tumour risks for *SDHB* mutation carriers at ages 60 years and 80 years were 23.9% (95% CI 20.9% to 27.4%) and 30.6% (95% CI 26.8% to 34.7%).

**Conclusions:**

Overall risks of clinically apparent tumours for *SDHB* mutation carriers are substantially lower than initially estimated and will improve counselling of affected families. Specific genotype–tumour risk associations provides a basis for novel investigative strategies into succinate dehydrogenase-related mechanisms of tumourigenesis and the development of personalised management for *SDHB/SDHC*/*SDHD* mutation carriers.

## Introduction

The succinate dehydrogenase (SDH) enzyme complex comprises four subunits (A–D), localises to the inner mitochondrial membrane and catalyses the oxidation of succinate during the Krebs cycle. Three subunit genes (*SDHB/SDHC/SDHD)* are among the most important susceptibility genes for the neuroendocrine tumours head and neck paraganglioma (HNPGL) and phaeochromocytoma and paraganglioma (PPGL).^1 2^ HNPGLs are derived from parasympathetic-derived ganglia, whereas PPGLs are derived from sympathetic ganglia and usually secrete catecholamines. HNPGL and PPGL have a strong genetic basis, with approximately 40% being associated with a germline mutation in one of at least 15 genes.^3^

Since the identification of the role of *SDHB/SDHC/SDHD* in inherited PPGL/HNPGL at the start of this century,^4–6^ testing for germline mutations in these genes has become part of standard medical practice. As testing became more widespread, it was revealed that mutations in *SDHC* and *SDHD* were associated with a higher risk of HNPGL than PPGL, with the reverse for *SDHB*.^7–10^ *SDHB* mutations are associated with a higher risk of malignancy and renal carcinoma than mutations in other subunits.^7 10 11^ Initially, the penetrance of *SDHB* mutations was estimated at ~70%–80%, and intensive surveillance programmes were recommended, but more recent estimates suggest the tumour risk is <50% in non-probands.^7 12–14^ The combination of malignancy risk with incomplete penetrance makes designing an optimal surveillance programme for asymptomatic *SDHB* mutation carriers difficult. More accurate predictions of life-time tumour risks and identification of subgroups with higher or lower risks would enable the development of personalised management and surveillance strategies.

Previously we reported the mutation spectrum and genotype–phenotype correlations in 358 patients with germline mutations in *SDHB* and *SDHD*.^10^ Here we report the results of analysis on an expanded cohort of germline *SDHB/SDHC/SDHD* mutation carriers and stratify missense mutations according to predicted effects on structure and function. We provide more accurate estimates of tumour-specific risks, confirm the mutation-specific phenotype of the *SDHD* p.Pro81Leu mutation and identify a novel candidate genotype–phenotype association of *SDHB* missense mutations with effects on *SDHB* protein stability.

## Materials and methods

### Patient cohort

The study sample consisted of men and women referred for *SDHB/SDHC/SDHD* mutation analysis in National Health Service diagnostic laboratories for a personal or family history of PPGL/HNPGL. Carriers of pathogenic mutations in other genes were excluded from this study. Clinical information was collected via a standard pro forma (see online supplementary figure 1), or clinical records, for research studies or a service evaluation study. Non-probands (those tested after a *SDHB*/*SDHC*/*SDHD* mutation was detected in their relative) were included except those with maternally inherited *SDHD* mutations who, because of a parent of origin effect, will have a tumour penetrance more similar to that of the general population.^15^ One proband, described previously,^16^ had a maternally inherited SDHD mutation. Affected individuals were diagnosed by routine clinical investigations. The work described was performed in accordance with the Declaration of Helsinki. All participants gave informed consent for genetic testing. The service evaluation study was approved by Birmingham Women’s Hospital R&D Office. A subgroup of patients (n=401) have been described previously^6 10 14 17–19^ with 358 included in Ricketts *et al*.^10^ We refer to patients as having ‘detailed clinical information’ if we have received a clinical information proforma or have access to their records to ascertain the presence or absence, and age of onset, of tumours. Patients without detailed clinical information were those for whom we had the genetic test report but insufficient extra information to include them in the penetrance analyses (they were censored at age zero). Clinical information was collected at the time of genetic testing and prior to baseline biochemical and imaging screening of asymptomatic gene carriers.

10.1136/jmedgenet-2017-105127.supp1 Supplementary file 1

### Molecular genetic analysis

*SDHB/SDHC/SDHD* mutations were detected by Sanger sequencing, next-generation sequencing assay^20^ or multiplex ligation-dependent probe amplification (MLPA) analysis (SALSA MLPA Kit P226; MRC-Holland, Amsterdam, The Netherlands) (details available on request).

*SDHB/SDHC/SDHD* sequence variants were classified as pathogenic/benign/variants of uncertain significance (VUS) by the reporting diagnostic laboratory. As methods of variant classification were not uniform between laboratories, all classifications were compared with the ClinVar classification,^21^ where available. Where there was a disagreement between the local laboratory classification and the ClinVar classification, the variant was described as VUS. This method was chosen to minimise the chance of underestimating penetrance of tumours because of inclusion of families without a true pathogenic variant. Tumour risks and genotype–phenotype correlations were calculated in individuals with variants considered to be pathogenic or likely pathogenic by the diagnostic laboratory (65 probands with a VUS were excluded from penetrance calculations).

### In silico protein structure analysis

The DUET and mCSM-PPI scoring systems^22–24^ were used to predict the structural consequences of missense mutations on protein stability and protein–protein affinity, respectively, using the models of *SDHB*, *SDHD* and the succinate complex (see supplementary information).

### Statistical analysis

Tumour risks were estimated by Kaplan-Meier analysis (*SDHB/SDHD/SDHC*). All statistical tests were performed using the programming language R unless otherwise stated.^25^ The ‘survfit’ function from the survival package was used for Kaplan-Meier survival analysis and penetrance calculations. The log-rank test was used to compare survival distributions between cohorts of different genotypes. To account for the non-random ascertainment of study participants with respect to their disease status, separate penetrance estimation analyses were carried out, in which we modelled the retrospective likelihood of the observed mutation status conditional on the disease phenotypes^26^ (online supplementary material). The analysis was carried out in the pedigree analysis software MENDEL.^27^ The retrospective cohort analysis was not used for *SDHD*, because it does not address the fact that the ascertainment of *SDHD* mutation carriers considered the parental origin of the mutation. The 0.05 level of significance was used for all tests, with Bonferroni correction for multiple comparisons where stated. All tests were two sided.

## Results

### Spectrum of SDHB/SDHC/SDHD mutations

Of 1832 patients referred for genetic testing due to a personal or family history of PPLG/HNPGL, 1093 were probands and 1227 had detailed clinical information available. Eight hundred seventy-six had a mutation in either *SDHB* (n=673, probands=275), *SDHC* (n=43, probands=26) or *SDHD* (n=160, probands=90) (see online supplementary figure 2).

### Copy number abnormalities (CNAs)

Forty-five probands had MLPA-detected single/multiple exon deletions or duplications in *SDHB* (n=36), *SDHC* (n=6) or *SDHD* (n=3) (see online supplementary table 1). Three of 36 *SDHB* probands with a CNA had a single or multiexon duplication. The proportion of *SDHB* CNAs that were whole gene deletions (5.6%), exon 1 deletions (49%) and exon 3 deletions (14%) was similar to that in other published cohorts,^8 28 29^ except for cohorts from the Netherlands where exon 3 deletions predominate.^30 31^ We found 83% (5/6) of *SDHC* CNAs were exon 6 deletions, and all three *SDHD* CNAs in our series were exon 4 deletions.

### Intragenic mutations

Three hundred and forty-four probands and 436 of their relatives harboured an intragenic mutation in *SDHB/SDHC/SDHD* (table 1). Forty-five intragenic mutations in 134 probands were reported previously.^10^ The ratio of mutation classes among probands was similar to that reported previously^10^ (44% missense, 15% nonsense, 13% splice-site, 15% frameshift, 0.5% inframe deletions and 12% large CNAs). There were a number of recurrent mutations, for example, *SDHB* splice-site c.72+1G>T and *SDHD* missense c.242C>T (p.Pro81Leu) mutations accounted for 20% of probands, and the 10 most common mutations accounted for 48% (see online supplementary figure 3).

**Table 1:** List of all intragenic mutations found in *SDHB*, *SDHC* and *SDHD*

Gene	Mutation	Amino acid change	No. of probands	LOVD ID^59^ or reference
*SDHB*	c.17_42dup26	p.Ala15ProfsX4	1	Jafri *et al* 14
*SDHB*	c.72+1G>A	Splice	3	LOVD ID SDHB_00171
*SDHB*	c.72+1G>C	Splice	1	Ricketts *et al* 10
*SDHB*	c.72+1G>T	Splice	36	LOVD ID SDHB_000065
*SDHB*	c.79C>T	p.Arg27X	7	LOVD ID SDHB_000006
*SDHB*	c.88delC	p.Gln30ArgfsX47	7	LOVD ID SDHB_000017
*SDHB*	c.118A>G	p.Lys40Glu	8	LOVD ID SDHB_000018
*SDHB*	c.136C>T	p.Arg46X	11	LOVD ID SDHB_000021
*SDHB*	c.137G>A	p.Arg46Gln	22	LOVD ID SDHB_000022
*SDHB*	c.141G>A	p.Trp47X	1	LOVD ID SDHB_000023
*SDHB*	c.166_170delCCTCA	p.Pro56TyrfsX5	2	LOVD ID SDHB_000025
*SDHB*	c.268C>T	p.Arg90X	8	LOVD ID SDHB_000001
*SDHB*	c.282_283insCTTA	p.Glu95LeufsX25	3	LOVD ID SDHB_000206
*SDHB*	c.286+2T>A	Splice	1	LOVD ID SDHB_000092
*SDHB*	c.286G>A	p.Gly96Ser	6	LOVD ID SDHB_000207
*SDHB*	c.287–1G>C	Splice	1	LOVD ID SDHB_000040
*SDHB*	c.292T>C	p.Cys98Arg	1	LOVD ID SDHB_000068
*SDHB*	c.296G>A	p.Gly99Asp	1	LOVD ID SDHB_000070
*SDHB*	c.297delC	p.Ser100LeufsX4	1	LOVD ID SDHB_000208
*SDHB*	c.298T>C	p.Ser100Pro	4	LOVD ID SDHB_000089
*SDHB*	c.302G>A	p.Cys101Tyr	1	LOVD ID SDHB_000041
*SDHB*	c.311delAinsGG	p.Asn104ArgfsX15	4	LOVD ID SDHB_000071
*SDHB*	c.325_335del11	p.Asn109LeufsX6	1	LOVD ID SDHB_000209
*SDHB*	c.338G>A	p.Cys113Tyr	3	LOVD ID SDHB_000210
*SDHB*	c.339_352del14	p.Cys113X	1	Not described
*SDHB*	c.343C>T	p.Arg115X	6	LOVD ID SDHB_000042
*SDHB*	c.379dupA	p.Ile127AsnfsX28	7	LOVD ID SDHB_000211
*SDHB*	c.380T>A	p.Ile127Asn	2	LOVD ID SDHB_000043
*SDHB*	c.380T>G	p.Ile127Ser	21	LOVD ID SDHB_000072
*SDHB*	c.418G>T	p.Val140Phe	3	LOVD ID SDHB_000095
*SDHB*	c.423+1G>A	Splice	3	LOVD ID SDHB_000047
*SDHB*	c.445C>T	p.Gln149X	1	Not described
*SDHB*	c.502dupC	p.Gln168ProfsX11	1	LOVD ID SDHB_000075
*SDHB*	c.526G>T	p.Glu176X	1	LOVD ID SDHB_000212
*SDHB*	c.552C>G	p.Tyr184X	1	Not described
*SDHB*	c.567_568delTG	p.Ala190LeufsX3	1	Not described
*SDHB*	c.587G>A	p.Cys196Tyr	7	LOVD ID SDHB_000054
*SDHB*	c.590C>G	p.Pro197Arg	11	LOVD ID SDHB_000002
*SDHB*	c.591delC	p.Ser198AlafsX22	1	LOVD ID SDHB_000003
*SDHB*	c.600G>T	p.Trp200Cys	17	LOVD ID SDHB_000098
*SDHB*	c.642+1G>T	Splice	1	Meyer-Rochow *et al* 60
*SDHB*	c.649C>T	p.Arg217Cys	1	LOVD ID SDHB_000086
*SDHB*	c.650G>T	p.Arg217Leu	1	LOVD ID SDHB_000159
*SDHB*	c.660dupT	p.Asp221X	1	LOVD ID SDHB_000057
*SDHB*	c.685_686ins13	p.Glu229AlafsX31	1	LOVD ID SDHB_000213
*SDHB*	c.688C>G	p.Arg230Gly	1	LOVD ID SDHB_000100
*SDHB*	c.688C>T	p.Arg230Cys	1	LOVD ID SDHB_000058
*SDHB*	c.689G>A	p.Arg230His	5	LOVD ID SDHB_000108
*SDHB*	c.724C>T	p.Arg242Cys	4	LOVD ID SDHB_000060
*SDHB*	c.725G>A	p.Arg242His	2	LOVD ID SDHB_000004
*SDHB*	c.745_748dupTGCA	p.Thr250MetfsX7	1	Jafri *et al* 14
*SDHC*	c.1A>T	Start codon mutation	3	LOVD ID SDHD_000006
*SDHC*	c.43C>T	p.Arg15X	1	LOVD ID SDHC_000013
*SDHC*	c.77+2dupT	Splice	1	Buffet *et al* 28
*SDHC*	c.148C>T	p.Arg50Cys	1	LOVD ID SDHC_000024
*SDHC*	c.345dupA	p.Ala116SerfsX2	1	Not described
*SDHC*	c.378T>G	p.Tyr126X	1	Not described
*SDHC*	c.380A>G	p.His127Arg	7	LOVD ID SDHC_000055
*SDHC*	c.397C>T	p.Arg133X	5	LOVD ID SDHC_000015
*SDHD*	c.1A>G	Start codon mutation	1	LOVD ID SDHD_000021
*SDHD*	c.14G>A	p.Trp5X	2	LOVD ID SDHD_000026
*SDHD*	c.33C>A	p.Cys11X	1	LOVD ID SDHD_000027
*SDHD*	c.36dupT	p.Ala13CysfsX56	1	Not described
*SDHD*	c.47_52+1del7	Splice	2	Not described
*SDHD*	c.53dupC	p.Leu19SerfsX50	1	Not described
*SDHD*	c.57delG	p.Leu20CysfsX66	1	LOVD ID SDHD_000080
*SDHD*	c.64C>T	p.Arg22X	2	LOVD ID SDHD_000012
*SDHD*	c.91dupA	p.Ile31AsnfsX38	1	Not described
*SDHD*	c.94_95delTC	p.Ala33IlefsX35	3	LOVD ID SDHD_000017
*SDHD*	c.98_108del11	p.Ala33GlyfsX32	1	Not described
*SDHD*	c.116_117insGATA	p.Pro41TyrfsX29	1	Not described
*SDHD*	c.144_145dupCA	p.Ile49ThrfsX38	3	Not described
*SDHD*	c.169+1G>A	Splice	2	LOVD ID SDHD_000132
*SDHD*	c.171delT	p.Gly58AlafsX28	1	Not described
*SDHD*	c.191_192delTC	p.Leu64ProfsX4	4	LOVD ID SDHD_000013
*SDHD*	c.205G>T	p.Glu69X	1	Not described
*SDHD*	c.242C>T	p.Pro81Leu	39	LOVD ID SDHD_000003
*SDHD*	c.242delC	p.Pro81ArgfsX5	1	LOVD ID SDHD_000151
*SDHD*	c.274G>T	p.Asp92Tyr	3	LOVD ID SDHD_000004
*SDHD*	c.276_278delCTA	p.Tyr93del	2	LOVD ID SDHD_000038
*SDHD*	c.296delT	p.Leu99ProfsX36	9	LOVD ID SDHD_000040
*SDHD*	c.325C>T	p.Gln109X	2	LOVD ID SDHD_000046
*SDHD*	c.341A>G	p.Tyr114Cys	1	LOVD ID SDHD_000007
*SDHD*	c.342T>A	p.Tyr114X	2	LOVD ID SDHD_000083

The 15 mutations labelled as ‘Not described’ have, to the best of our knowledge, not been published elsewhere.

### Clinical presentation

Of 297 probands with a *SDHB*/*SDHC/SDHD* mutation and detailed clinical information, the presenting features were predominantly PPGL and HNPGL, though the frequency varied by gene (see  online supplementary table 3). Three hundred and seventy-four of the 454 non-proband SDH mutation carriers with detailed clinical information were asymptomatic at testing.

### Age-related tumour risks

Tumour risks were estimated by Kaplan-Meier analysis for all *SDHB*/*SDHD*/*SDHC* mutation carriers, probands only and non-probands only for *SDHD* non-proband carriers only those with paternally inherited mutations). For *SDHB*, retrospective cohort analysis was also performed using all samples combined (probands and non-probands), probands only and using non-probands only.

**Figure 1:**
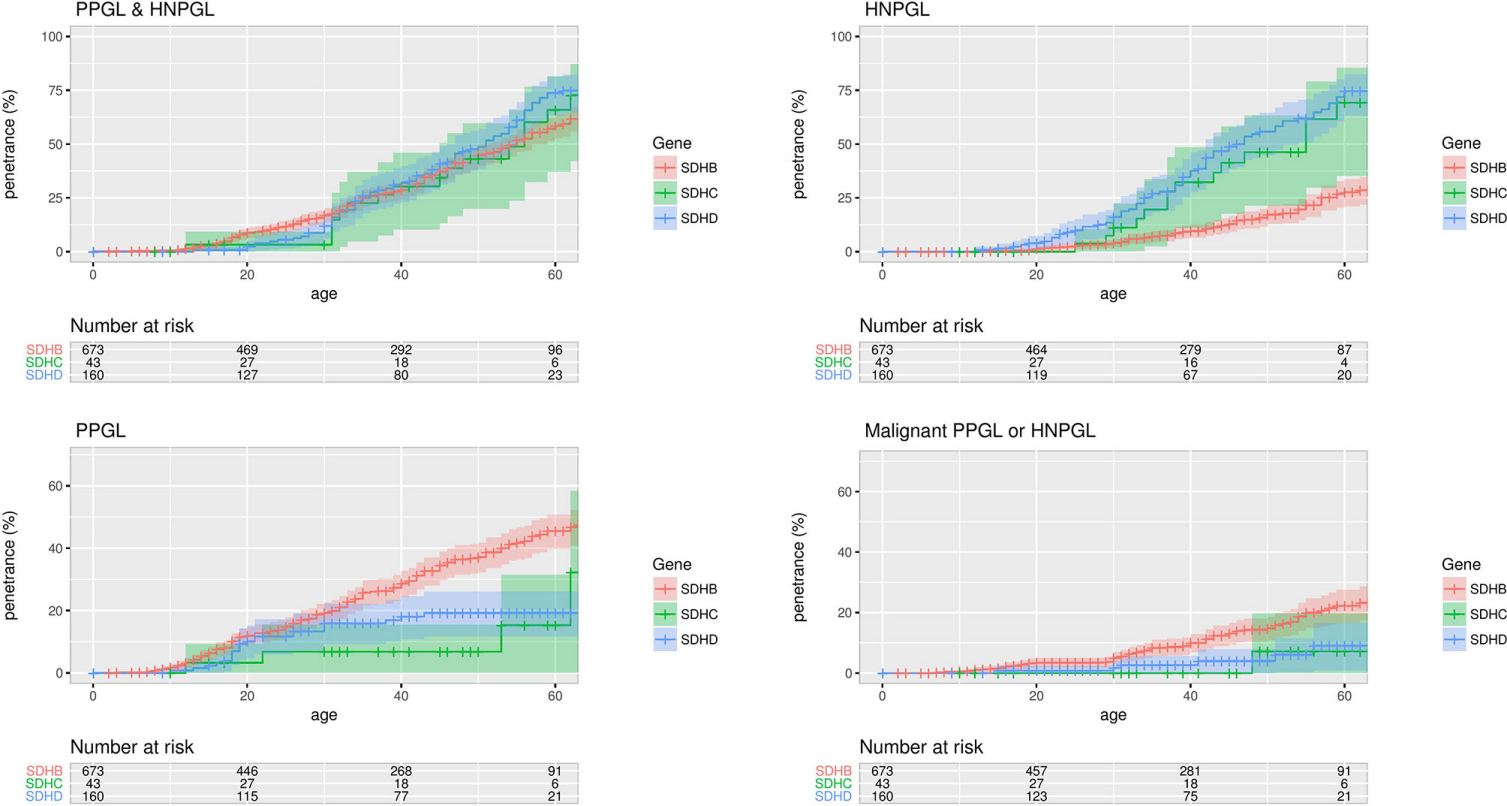
Penetrance of for clinically diagnosed disease in proband and non-proband *SDHB*, *SDHC* and *SDHD* mutation carriers with 95% CI shaded. HNPGL, head and neck paraganglioma; PPGL, phaeochromocytoma and paraganglioma.

Kaplan-Meier analysis (and log-rank testing) of probands and non-probands (figure 1) revealed higher penetrance for symptomatic tumours in *SDHD* than in *SDHB* mutation carriers (P=0.032), increased age-related risk of HNPGL in *SDHD* than *SDHB* mutation carriers (P<0.0001) and higher risk of PPGL in *SDHB* than *SDHD* (P=0.00019). *SDHB* carriers were more likely to develop malignant disease (P=0.0043). These findings remained significant when the previously reported 358 patients^10^ were excluded from the analysis (data not shown).

In 34 *SDHC* mutation carriers with detailed clinical information, 19 were clinically affected (15 HNPGL, 3 PPGL and 1 had both HNPGL and PPGL). One patient with HNPGL had local spread and malignant features. Age-related risks of symptomatic PPGL/HNPGL in *SDHC* were similar to that of *SDHD.* Compared with *SDHB* mutation carriers, *SDHC* carriers had a lower risk of PPGL (P=0.02 and P=0.06 before and after Bonferroni correction) and a higher risk of HNPGL (P<0.001 after Bonferroni correction for three comparisons, log-rank test) (figure 1).

Overall tumour risks will be inflated by including both probands and non-probands (as probands always have the disease) so we re-estimated the cumulative risks in non-probands only. Using Kaplan-Meier analysis, the estimated risk of PPGL/HNPGL at age 60 years in *SDHB*, *SDHD* and *SDHC* mutation positive non-probands was 21.8% (95% CI 15.2% to 27.9%), 43.2% (95% CI 25.4% to 56.7%) and 25% (95% CI 0% to 57.0%), respectively. Nine non-probands (all *SDHB*) developed malignant disease, and the risk of malignant disease at age 60 years in non-proband *SDHB* mutation carriers was 4.2% (95% CI 1.1% to 7.2%).

**Figure 2:**
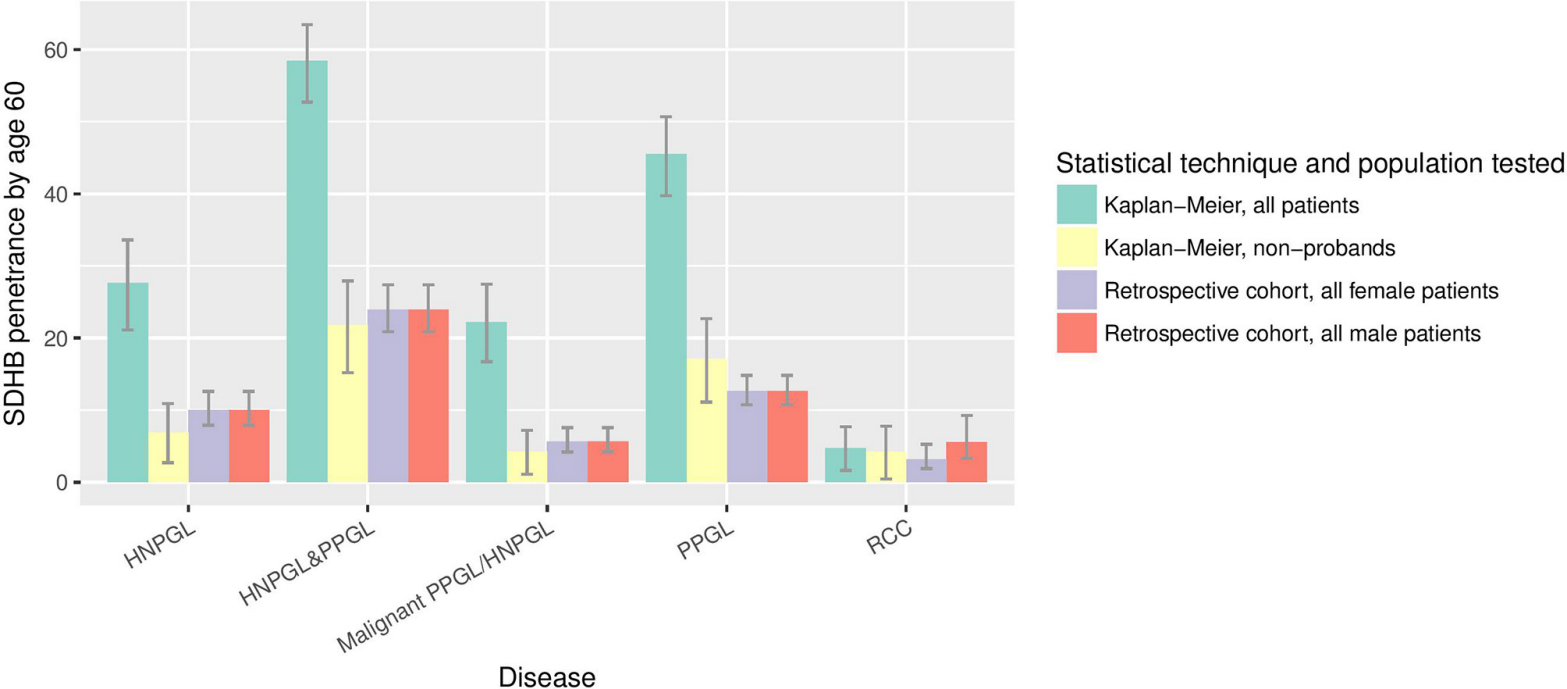
Penetrance of clinical disease in *SDHB* mutation carriers by age 60 years, as calculated by different statistical techniques and in different subpopulations. HNPGL,  head and neck paraganglioma; PPGL, phaeochromocytoma and paraganglioma; RCC, renal cell carcinoma.

As expected, estimates of penetrance from the retrospective cohort analysis of a combined sample of probands and non-probands were lower than the above estimates from Kaplan-Meier analysis as the former method provides some adjustment for ascertainment bias. Under the retrospective cohort analysis, the predicted penetrance of PPGL/HNPGL in *SDHB* mutation carriers (probands and non-probands) by age 60 years and age 80 years was 23.9% (95% CI 20.9% to 27.4%) and 30.6% (95% CI 26.8% to 34.7%), respectively (see figure 2), similar to the Kaplan-Meier estimates in non-probands.

Male *SDHB* mutation carriers have a higher age-related penetrance of PPGL/HNPGL (P=0.0034) and PPGL (P=0.0079), compared with women (see online supplementary figure 4), as calculated by Kaplan-Meier analysis and log-rank testing.

### Other tumours

Previously,^10^ we reported 12/358 patients had renal tumours (11 *SDHB* and 1 *SDHD*), and the updated cohort contains an additional four cases (all *SDHB*). Of 751 patients with detailed clinical information (584 *SDHB*, 33 *SDHC* a*nd* 134 *SDHD*), 15 (2.6%) *SDHB* carriers and 1 (0.7%) *SDHD* carrier had a renal tumour. The risk of developing a renal tumour by age 60 years in *SDHB* mutation carriers was 4.2% (95% CI 0.46% to 7.8%) by Kaplan-Meier analysis of non-probands, 4.71% (95% CI 1.65% to 7.7%) by Kaplan-Meier analysis of all *SDHB* mutation carriers and 5.6%/3.2% (male/female) by retrospective cohort analysis of all *SDHB* mutation carriers. No further thyroid tumours were found beyond the three described previously in *SDHB* mutation carriers,^10^ giving an estimated penetrance of thyroid tumours by age 60 years of 1.5% (95% CI 0.0% to 3.1%) (calculated by Kaplan-Meier analysis of all SDHB mutation carriers). Other rare tumours included: (A) *SDHB* carriers: pituitary adenoma, parathyroid adenoma and pulmonary carcinoid tumour; (B) *SDHC*: pituitary adenoma, gastrointestinal stromal tumour (GIST); and (C) *SDHD:* a pituitary tumour.

#### Genotype–phenotype correlations

##### Structural prediction analysis

*SDHB* and *SDHD* missense mutations were analysed using DUET^24^ to predict their effect on protein stability and mCSM-PPI to predict their effect on succinate complex formation^22^ (table 2).

**Table 2:** DUET score for all *SDHB* and *SDHD* missense mutations described in this cohort and for rare missense variants with no or unknown pathogenicity

Mutation	Allele frequency	mCSM stability (Kcal/mol)	DUET (Kcal/mol)	Predicted effect
SDHB	–	–	–	–
p.Lys40Glu	Unknown	−1.741	−1.774	Destabilises protomer and complex
p.Arg46Gln	0.000008238	−0.997	−1.02	Destabilises protomer
p.Gly96Ser	0.000008381	−1.024	−1.09	Destabilises protomer; positive phi glycine; affect metal binding
p.Cys98Arg	Unknown	0.217	0.316	Affect metal binding; destabilse complex
p.Gly99Asp	Unknown	−0.989	−1.216	Destabilises protomer and complex; affect metal binding
p.Ser100Pro	Unknown	−0.226	−0.368	Affect metal binding; destabilse complex
p.Cys101Tyr	Unknown	−1.06	−1.651	Destabilises protomer; affect metal binding
p.Cys113Tyr	Unknown	−0.974	−1.467	Destabilises protomer and complex; affect metal binding
p.Ile127Asn	Unknown	−3.088	−3.213	Destabilises protomer. Mildly destabilises complex
p.Ile127Ser	Unknown	−3.669	−3.856	Destabilises protomer
p.Val140Phe	Unknown	−1.233	−1.353	Destabilises protomer. Mildly destabilises complex
p.Cys196Tyr	Unknown	−1.407	−1.77	Destabilises protomer and complex; affect metal binding
p.Pro197Arg	Unknown	−0.954	−0.784	Destabilises protomer; mildly destabilises complex; affect metal binding; loss of conformational restraint
p.Trp200Cys	Unknown	−1.442	−1.205	Destabilises protomer and complex
p.Arg217Cys	Unknown	−1.916	−1.948	Destabilises protomer and complex
p.Arg217Leu	Unknown	−1.031	−0.879	Destabilises protomer and complex
p.Arg230Gly	Unknown	−1.848	−2.45	Destabilises protomer and complex
p.Arg230Cys	0.000008252	−1.739	−1.836	Destabilises protomer and complex
p.Arg230His	Unknown	−1.903	−2.133	Destabilises protomer
p.Arg242Cys	Unknown	−1.386	−1.619	Destabilises protomer; mildly destabilises complex; affect metal binding
p.Arg242His	0.00002471	−1.948	−2.035	Destabilises protomer; mildly destabilises complex; affect metal binding
SDHD	–	–	–	–
p.Pro81Leu	Unknown	−0.297	0.036	Destabilising transmembrane
p.Asp92Tyr	Unknown	−0.907	−0.868	Destabilising transmembrane
p.Tyr114Cys	Unknown	0.236	0.388	Stabilising

Mutations with no or unknown pathogenicity				
Mutation	Allele frequency	mCSM	DUET	Predicted effect
SDHB	–	–	–	–
p.Ala3Gly	0.00436	−0.28	−0.244	Neutral
p.Thr17Ala	Unknown	−0.943	−0.82	Destabilising
p.Cys22Ser	0.0001331	0.119	0.135	Neutral
p.Arg27Gly	0.00004183	−0.229	−0.208	Neutral
p.His57Arg	0.0007249	−0.238	0.011	Neutral
p.Ser100Phe	Unknown	−0.226	−0.368	Neutral
p.Ser163Pro	0.01254	−0.136	−0.03	Introduction of a conformational restraint
SDHD	–	–	–	–
p.Gly12Ser	0.007268	−0.377	−0.134	Altered localisation
p.Arg17Leu	Unknown	0.549	0.481	Mildly stabilising
p.Ile40Val	0.00003295	−0.461	−0.182	Neutral
p.Glu42Ala	Unknown	−0.255	−0.017	Neutral
p.His50Arg	0.006515	−0.4	−0.155	Altered localisation
p.Pro87His	Unknown	−0.571	−0.442	Neutral
p.His145Asn	Unknown	−0.055	0.018	Neutral

Allele frequencies are as reported in the ExAC database (Exome Aggregation Consortium, Cambridge, Massachusetts, USA), all ethnicities, accessed 17 June 2017^61^

*SDHB/SDHD* variants with no or uncertain pathogenicity were predicted by DUET to have little effect on either protein stability and protein complex formation (average ΔΔG −0.15 Kcal/mol) in contrast to disease associated mutations (average ΔΔG −1.41 Kcal/mol) (P<0.001 two-tailed t-test).

**Figure 3:**
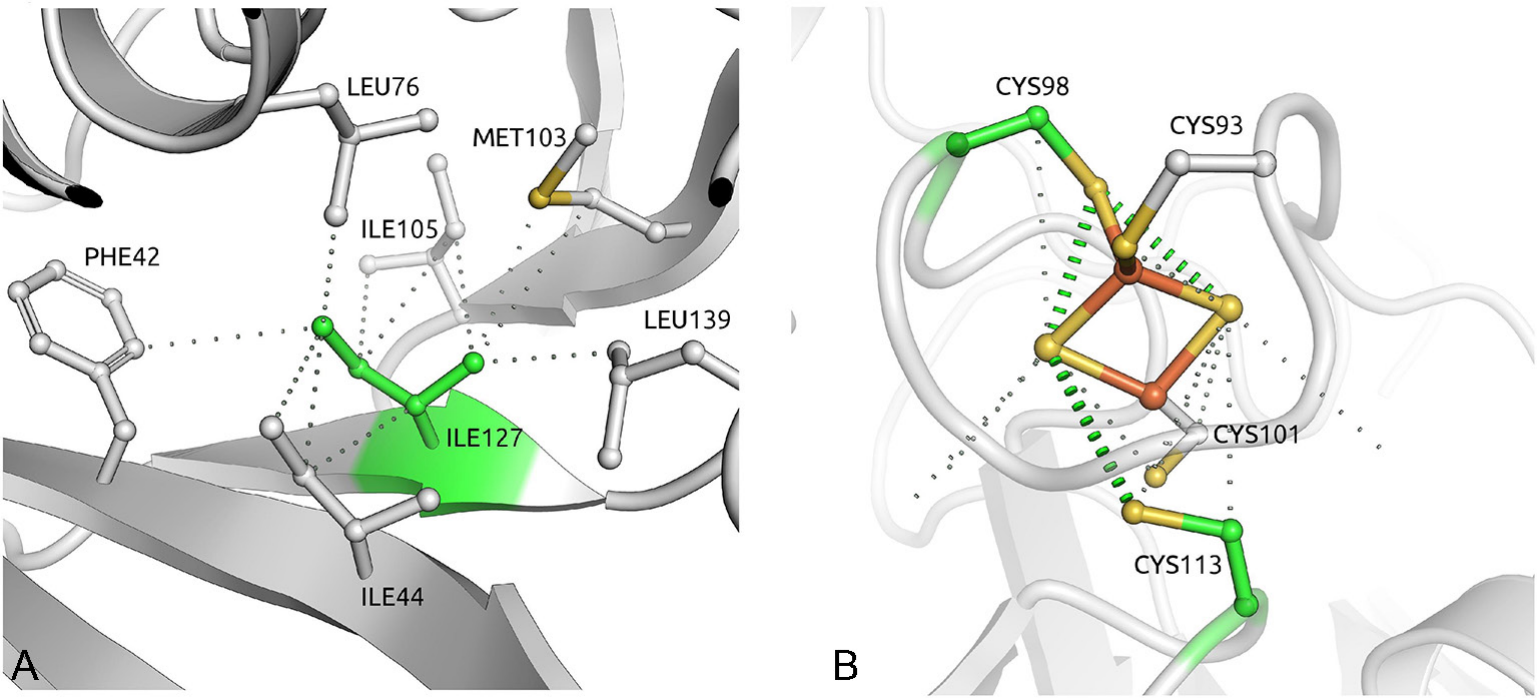
(A) The isoleucine reside at position 127 of *SDHB* is buried in the middle of the structure, making a network of strong intramolecular hydrophobic interactions. Mutation to serine would introduce a polar residue and disrupt all of these important contacts. (B) One of three iron–sulphur clusters in SDHB, coordinated by four cysteine residues. Four of these (Cys98, Cys101, Cys113 and Cys196) are mutated in our cohort.

Of the 21 disease-associated *SDHB* missense mutations, 20 are predicted to be destabilising, 16 are predicted to destabilise the complex and 4 (p.Cys98Arg, p.Cys101Tyr, p.Cys113Tyr and p.Cys196Tyr) are in metal coordinating cysteines. The most destabilising *SDHB* missense mutation, p.Ile127Ser, affected an isoleucine residue buried deep in the protein with a strong network of intramolecular hydrophobic interactions^32^ (figure 3A). The three SDHB mutations not predicted to alter protein stability were all predicted to affect the coordination of an iron–sulphur cluster, either directly or by affecting neighbourhood residues (figure 3B).

#### Structure–phenotype correlations and mutation specific phenotypes

**Figure 4:**
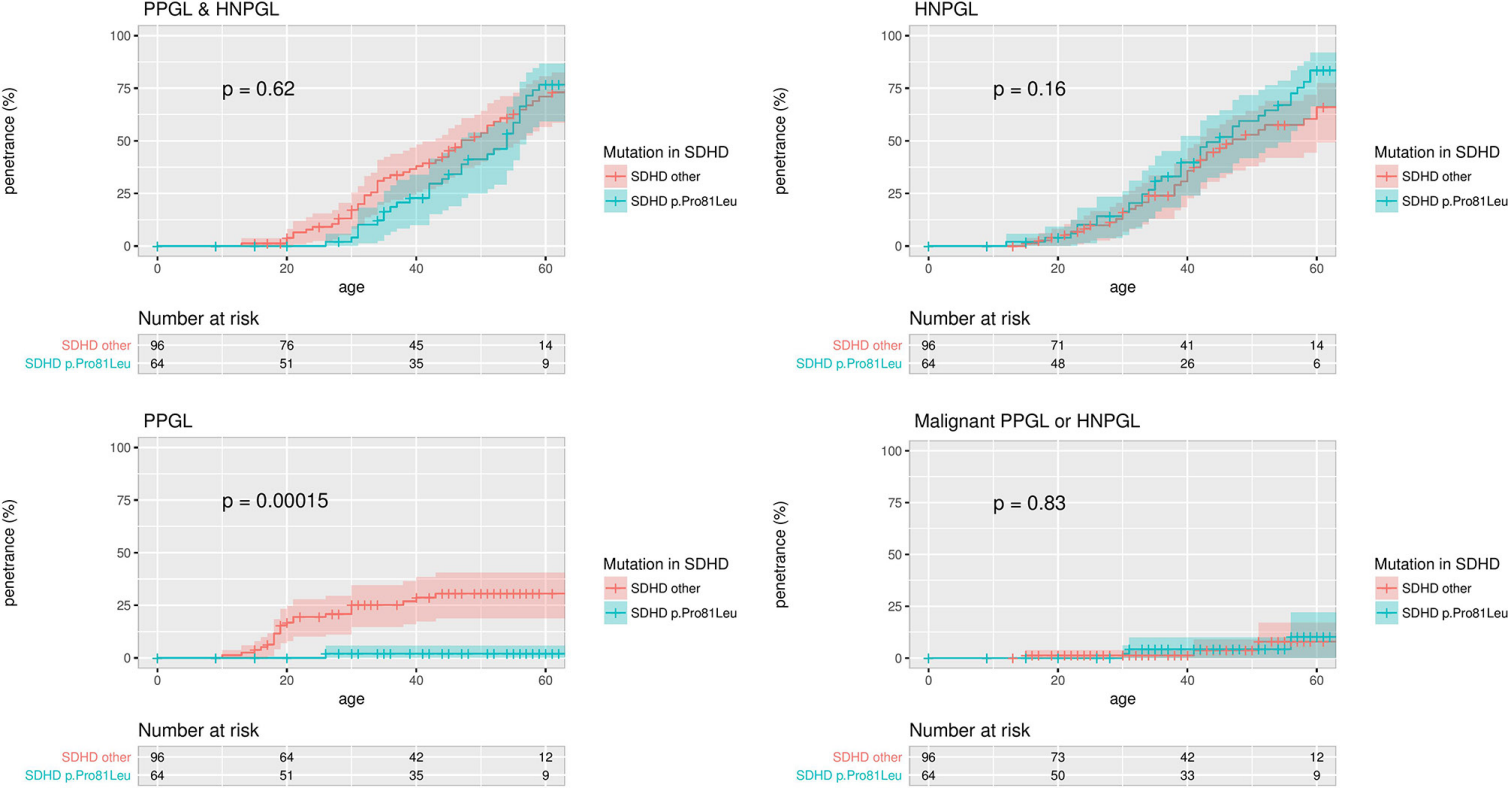
Penetrance of clinical disease in proband and non-proband *SDHD* p.Pro81Leu mutation carriers versus all other *SDHD* mutation carriers with 95% CI marked. P values are for the log-rank test comparing the survival distributions of *SDHD* p.Pro81Leu and all other *SDHD* mutation carriers. HNPGL, head and neck paraganglioma; PPGL, phaeochromocytoma and paraganglioma.

We confirmed our previous observation^10^ that the *SDHD* p.Pro81Leu phenotype is distinct from that of other *SDHD* mutation carriers, with a low PPGL risk (figure 4). Of 53 individuals with detailed clinical information, 15 were asymptomatic, 37 had HNPGL (two metastatic) and 1 had PPGL (described previously by Yeap *et al*).^16^ The p.Pro81Leu mutation is predicted to have a very mild effect on protein stability. Excluding the cases originally analysed by Ricketts *et al* 10 to create a replication cohort confirmed the lower risk of PPGL in *SDHD* p.Pro81Leu mutation carriers versus other *SDHD* mutation carriers (P=0.031, data not shown).

**Figure 5:**
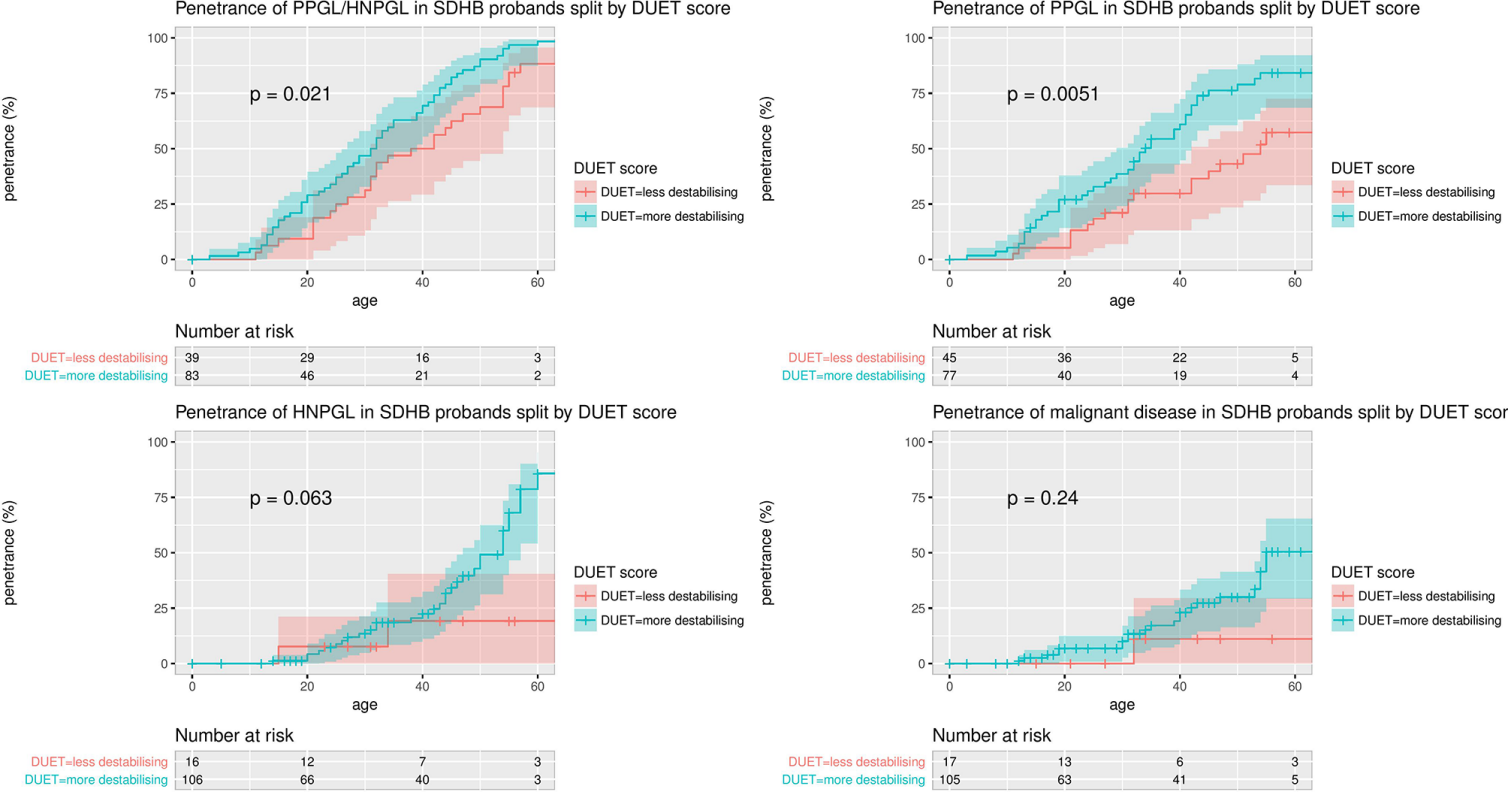
Penetrance of clinical disease in *SDHB* missense mutation carrier probands, split by with more stabilising (more negative) and less destabilising (less negative) DUET scores. The maximally selected rank statistics from the ‘maxstat’ R package was used to find the optimum cutpoint for DUET score, and the number of probands in the DUET score groups for each analysis is displayed in ‘Number at risk’ tables below each plot. The optimum cutpoints for DUET score were −1.21 for PPGL risk and for PPGL/HNPGL risk, −0.88 for HNPGL risk and −1.02 for malignancy risk. HNPGL, head and neck paraganglioma; PPGL, phaeochromocytoma and paraganglioma.

There were no differences in age-related risks of PPGL/HNPGL between missense and truncating variants (data not shown). However, there was a higher overall penetrance for clinical disease (all tumour risk) (P=0.0047) and PPGL risk (P=0.00024) in p.Ile127Ser mutation carriers (the missense mutation with the highest DUET score for predicted protein instability) compared with other missense mutations. Furthermore, using the surv_cutpoint() function in R, which is designed to use the maximally selected rank statistics from the ‘maxstat’ R package to find the optimum cutpoint for continuous variables, the PPGL/HNPGL and PPGL risks were significantly higher for those missense mutations with the most destabilising DUET scores (PPGL/HNPGL penetrance: P=0.0086; PPGL penetrance: P=0.00025). The significance of these differences was preserved when calculated for probands only (PPGL/HNPGL penetrance: P=0.021; PPGL penetrance: P=0.0051, see figure 5).

## Discussion

To our knowledge, this is the largest series of *SDHB*/*SDHC*/*SDHD* mutation carriers yet reported (see  online supplementary tables 2 and 4) and the previous largest cohort from Buffet *et al* 28 (363 probands, 269 with *SDHB/C/D* mutations), which did not report genotype–phenotype correlations. Our large-scale comprehensive genotype–phenotype assessments uncovered a series of novel observations:

### Gene-specific and mutation-specific differences in penetrance and expression

We unequivocally confirmed that *SDHD* mutation carriers had a higher overall penetrance for symptomatic tumours and a higher risk of HNPGL compared with *SDHB*, whereas *SDHB* mutation carriers had a higher risk of malignancy and were significantly more likely to develop PPGL. We have confirmed the findings of Jochmanova *et al* 33 that male *SDHB* mutation carriers are at higher risk of disease than females (calculated by Kaplan-Meier analysis and log-rank testing). The reason for this observation is not clear. Although it is possible that because asymptomatic female at risk relative might be more likely to come forward for predictive genetic testing than males we note that in our cohort, there were more male than female probands with *SDHB* mutations (153 male, 110 female), and there were equal numbers of male and female non-probands with *SDHB* mutations (198 male, 201 female). Of the unaffected at risk relatives without a mutation (relatives of SDHB/D/C mutation carriers), 130 were female and just 103 were male.

The phenotype associated with *SDHC* mutations has not been well defined. Early reports described benign HNPGLs,^5 34 35^ but more recent studies have also identified extra-adrenal paragangliomas and more invasive tumours. Bickmann *et al* 36 and Bourdeau *et al* 37 both describe *SDHC* p.Arg133X mutation carriers (a mutation that is also common in our cohort) presenting with extra-adrenal paragangliomas and HNPGLs, both benign and malignant. Our findings of *SDHC* mutation carriers with extra-adrenal paragangliomas, pheochromocytoma and a case of HNPGL with malignant features are consistent with similar tumour risks with *SDHC* and paternally inherited *SDHD* mutations (Lefebvre and Foulkes^38^ recommend similar tumour screening for *SDHC* and *SDHD* mutation carriers).

We replicated our previous finding that *SDHD* p.Pro81Leu mutation carriers manifest almost exclusively with HNPGL, while other *SDHD* mutation types predispose to both HNPGLs and PPGLs. From a clinical perspective, it can be proposed that *SDHD* p.Pro81Leu mutation carriers do not need intensive imaging for PPGL. Although the risk of PPGL in *SDHD* p.Pro81Leu mutation carriers was not zero, the single patient with a phaeochromocytoma was highly unusual^16^ and was the only example of tumour development after maternal inheritance of a *SDHD* mutation. Interestingly, all of the rare cases of tumours in individuals with maternally inherited *SDHD* mutations have been phaeochromocytomas and none have had a HNPGL.^16 39 40^ *SDHD* has a key role in anchoring the SDH complex to the inner mitochondrial membrane, and a truncating *SDHD* mutation would inactivate the function of the entire SDH complex and be predicted to lead to disordered signalling in the hypoxic gene response pathway and to epigenetic abnormalities resulting from inhibition of enzymes such as prolyl-hydroxylases and ten-eleven translocation enzymes.^41–44^ The *SDHD* p.Pro81Leu mutation is predicted not to cause protein instability but to interfere with ubiquinone metabolism/electron transport by changing the folding of helix 1S and by destroying a ubiquinone binding site.^10 45^ The small risk of PPGL with the p.Pro81Leu mutations suggests that PPGL and HNPGL result from impairment of different aspects of the function of the SDH complex.

Our in silico analysis of the structural effects of *SDHB* missense mutations revealed a novel genotype–phenotype association in which the missense mutations predicted to have the greatest protein destabilising effects were associated with a higher penetrance and risk of PPGL. If these findings are confirmed, they could be used to stratify tumour surveillance programmes according to individual mutation risks. They could complement other methods, such as tumours studies and in vitro functional studies in human tissues or model organisms such as yeast,^46^ to elucidate the molecular mechanisms and clinical significance of the mutation.

### Other tumours

We also found a variety of rarer tumour types in individuals with *SDHB/SDHC/SDHD* mutations, including renal cell carcinoma (RCC), GIST, thyroid and pituitary tumours.^7 10 47–51^ The highest risk for RCC is in *SDHB* mutation carriers (though they can occur in *SDHD* and *SDHC* mutation carriers), and we estimated the risk of RCC by age 60 years in *SDHB* carriers is 4.7% (95% CI 1.6% to 7.7%) by Kaplan-Meier analysis of probands and non-probands and 5.63%/3.18% (male/female risk) by retrospective cohort analysis.

Although the risk of renal tumours is smaller than for PPGL, if abdominal imaging is being performed for PPGL, it is straightforward to incorporate renal imaging. Our results suggest that thyroid imaging is not indicated if asymptomatic.

### Penetrance in non-probands

Accurate knowledge of the natural history of a disease is required for optimum management. For rare diseases, there may be limited numbers of affected individuals, and ascertainment bias in research studies can result in over-representation of extreme phenotypes with overestimation of disease risks. Interestingly, with wider application of *SDHB/C/D* genetic testing, penetrance estimates for *SDHB* mutation carriers have declined. Original methods used Kaplan-Meier analysis of both probands and non-probands and produced penetrance estimates such as 75% by age 50 years,^7^~55% by age 50 years^12^ and 50% by age 50 years.^10^ Subsequently, analyses have been published that have controlled for ascertainment bias by excluding probands, producing estimates more like 20% by age 50 years.^14 33^ An alternative approach has been to study a single large family so that the majority of patients analysed are non-probands, producing penetrance estimates of 35% by age 40 years^52^ and 26% by age 48.^53^ More recently, the use of maximum likelihood methods to control for ascertainment bias has resulted in estimates of penetrance as low as 13%,^13^ 9%^54^ and 21%^55^ at age 50 years.

The above cohorts have ranged in size from 15 to 344 *SDHB* mutation carriers. To better understand the prognosis for in individuals undergoing predictive testing, we tested a large (n=584 with detailed clinical information) cohort of *SDHB* mutation carriers, adjusting for ascertainment bias using two approaches. We estimated overall clinical penetrance and tumour-specific risks by Kaplan-Meier analysis after excluding probands and compared the estimates with those obtained by retrospective cohort analysis in *SDHB* mutation carriers. We estimated clinical disease penetrance in non-proband *SDHB* mutation carriers at 50 years, 60 years and 80 years to be 16%, 22% and 44%, respectively, and these were similar with the retrospective cohort analysis estimates: cumulative risk of 24% for PPLG/HNPGL by age 60 years and 31% by age 80 years. One limitation to our analysis was that the unavailability of pedigree structures that would have allowed us to look for potential parent-of-origin effects and to fully adjust for ascertainment.

The recent demonstrations of lower tumour risks raise important questions regarding follow-up for asymptomatic *SDHB* mutation carriers. Blood/urine biochemical analysis to detect metanephrines is inexpensive, but the risk of non-secretory and malignant PPGL/HNPGL and renal and GIST tumours would argue for regular imaging. The Endocrine Society guidelines^56^ recommend annual biochemistry with urine or plasma metanephrines and 2 yearly cross-sectional imaging using either CT/MRI of skull base, neck, thorax abdomen and pelvis. It should be noted that the our tumour risk estimates are for clinically apparent disease and are censored at the time of genetic testing. Subsequently, asymptomatic mutation carriers will have undergone biochemical and radiological surveillance and, though this information was not available, some may have been found to have an asymptomatic tumour. Thus, in a study of 30 SDHx mutation carriers,^57^ a tumour was detected in one patient after 1 year follow-up from normal baseline imaging. Tufton *et al* 58 describe a cohort of 65 asymptomatic *SDHB* mutation carriers undergoing surveillance by MRI. They found that 25% of these patients had a likely SDH-related tumour identified after baseline imaging or after a further 2–6 years of screening. The penetrance estimates for clinically apparent tumours in our study will be lower than those estimated in studies where patients have completed comprehensive screening and follow-up but are comparable with those from other large cohort studies and are consistent with more recent, although smaller, studies (see above) that have concluded that *SDHB* mutations are not associated with a high lifetime risk for clinically apparent tumours. Although the non-inclusion of asymptomatic screen-detected lesions might be viewed as a limitation of the study design, it does avoid the uncertainties regarding whether asymptomatic tumours would become symptomatic, and the risk estimates for clinically apparent tumours are highly relevant to patients wishing to know their lifetime risks of having a symptomatic tumour.

In the light of the robust data from this and other studies demonstrating lower than previously estimated tumour risks in *SDHB* non-proband mutation carriers, there is a pressing need to continue to systematically collect data on the outcome of surveillance programmes. It is also highly relevant to develop strategies to enable patient stratification (eg, individuals at high risk because of specific mutations or family history/genetic modifiers) and to enable non-invasive early detection of tumours.
