# Correction: *Tumour risks and genotype-phenotype correlations associated with germline variants in the succinate dehydrogenase subunit genes SDHB, SDHC, and SDHD*

**DOI:** 10.1136/jmedgenet-2017-105127corr1

**Published:** 2018-11-22

Andrews KA, Ascher DB, Pires DEV, *et al*. Tumour risks and genotype-phenotype correlations associated with germline variants in the succinate dehydrogenase subunit genes *SDHB, SDHC*, and *SDHD. J Med Genet* 2018;55:384–94. doi: 10.1136/jmedgenet-2017-105127.

In this paper, Kaplan Meier analysis estimates of the combined penetrance of phaeochromocytoma and paraganglioma (PPGL) and head and neck paraganglioma (HNPGL) was incorrectly reported. This was because of an error importing data into the statistical software R.

The following penetrance estimates were affected:

­

- The penetrance of PPGL/HNPGL in SDHB non-probands by age 60, reported as ‘21.8% (95% CI 15.2% to 27.9%)’, should read ‘22.5% (95%CI 15.9% to 28.6%)’ (see sentence 9 of abstract, sentence 2 of paragraph 4 under the section head ‘Age-related tumour risks’ of the results, and figure 2).
- The penetrance of PPGL/HNPGL in SDHD non-probands by age 60, reported as ‘43.2% (95% CI 25.4% to 56.7%)’, should read ‘50.0% (95% CI 31.2% to 63.7%)’ (see sentence 9 of abstract, and sentence 2 of paragraph 4 under the section head ‘Age-related tumour risks’ of the results).
- The penetrance of PPGL/HNPGL in SDHB probands and non-probands by age 60, reported as ‘58.4%’, should read ‘60.2%’ (see figures 1 and 2).
- The penetrance of PPGL/HNPGL in SDHC and SDHD probands and non-probands by age 60, reported as ‘66%’, and ‘74%’ respectively, should read ‘75%’, and ‘79%’ (see figure 1, panel entitled ‘PPGL & HNPGL’).
- The p value from log-rank testing comparing penetrance of PPGL/HNPGL in SDHD versus SDHB, reported as ‘0.032’, should read ‘0.0017’ (see sentence 1 of paragraph 2 under the section head ‘Age-related tumour risks’ of the results)
- The p value from log-rank testing comparing penetrance of PPGL/HNPGL in male versus female SDHB mutation carriers, reported as ‘0.0034’, should read ‘0.0087’ (see sentence 1 of paragraph 6 under the section head ‘Age-related tumour risks’ of the results, and online supplementary figure 3).
- The p value from log-rank testing comparing penetrance of PPGL/HNPGL in SDHD p.Pro81Leu versus other SDHD, reported as ‘0.62’, should read ‘0.81 (see figure 4, panel entitled ‘PPGL & HNPGL’).
- The p value from log-rank testing comparing penetrance of PPGL/HNPGL in SDHB p.Ile127Ser versus other SDHB missense mutations, reported as ‘0.0047’, should read ‘0.02’ (see sentence 2 of paragraph 2 under the section head ‘Structure-phenotype correlations and mutation specific phenotypes’ of the results).
- The penetrance of PPGL/HNPGL in SDHB non-probands by age 80, reported as ‘44%’, should read ‘39%’ (see sentence 4 of paragraph 2 under the section head ‘Penetrance in non-probands’ of the discussion).

­

Neither the statistical significance of any result nor the conclusions drawn in the paper are affected by this error. Revised versions of figure 1, 2, 4 and supplementary figure 4 are also published along this correction article.

**Figure 1:**
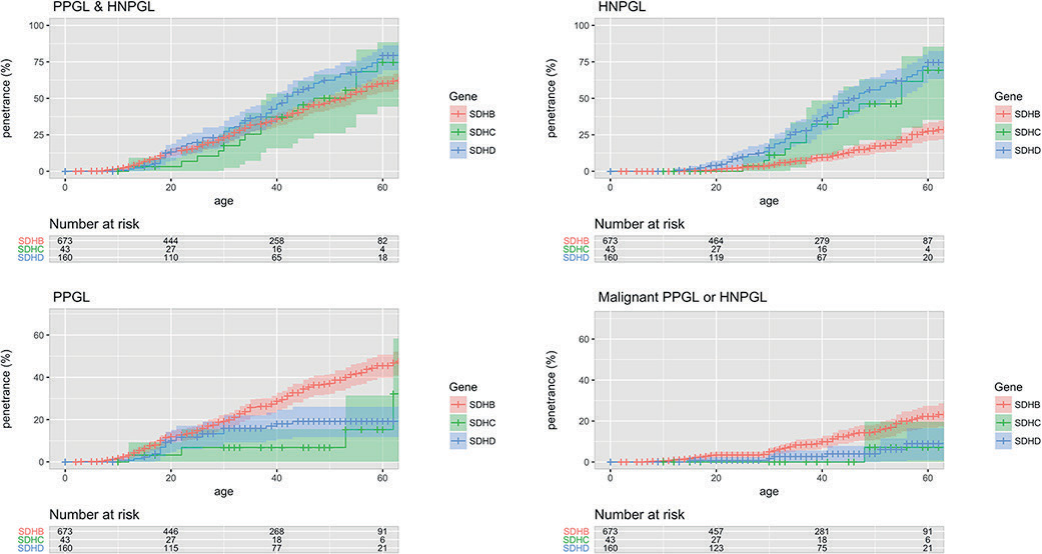
Penetrance of for clinically diagnosed disease in proband and non-proband SDHB, SDHC and SDHD mutation carriers with 95% CI shaded. HNPGL, head and neck paraganglioma; PPGL, phaeochromocytoma and paraganglioma.

­

**Figure 2:**
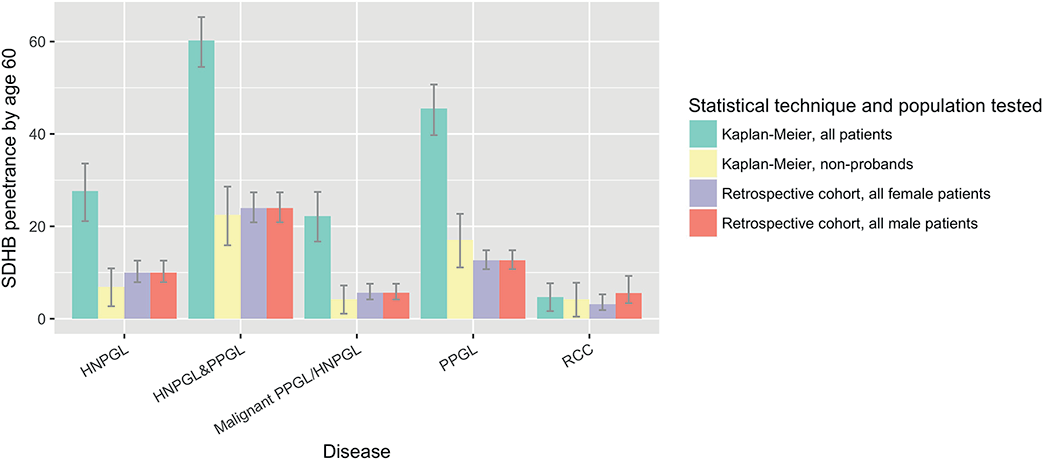
Penetrance of clinical disease in SDHB mutation carriers by age 60 years, as calculated by different statistical techniques and in different subpopulations. HNPGL, head and neck paraganglioma; PPGL, phaeochromocytoma and paraganglioma; RCC, renal cell carcinoma.

­

**Figure 3:**
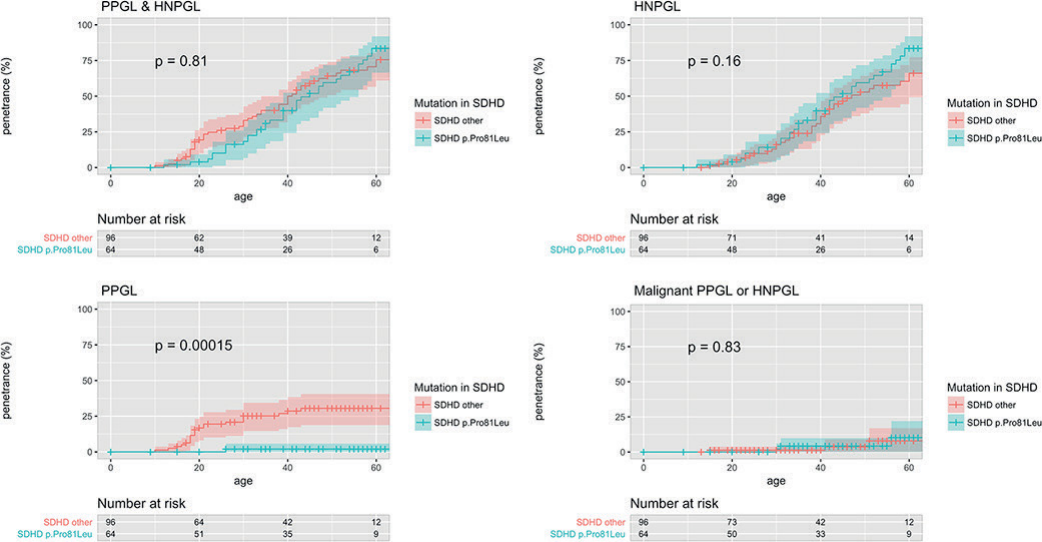
Penetrance of clinical disease in proband and non-proband SDHD p.Pro81Leu mutation carriers versus all other SDHD mutation carriers with 95% CI marked. P values are for the log-rank test comparing the survival distributions of SDHD p.Pro81Leu and all other SDHD mutation carriers. HNPGL, head and neck paraganglioma; PPGL, phaeochromocytoma and paraganglioma.

­

­

**Figure 4:**
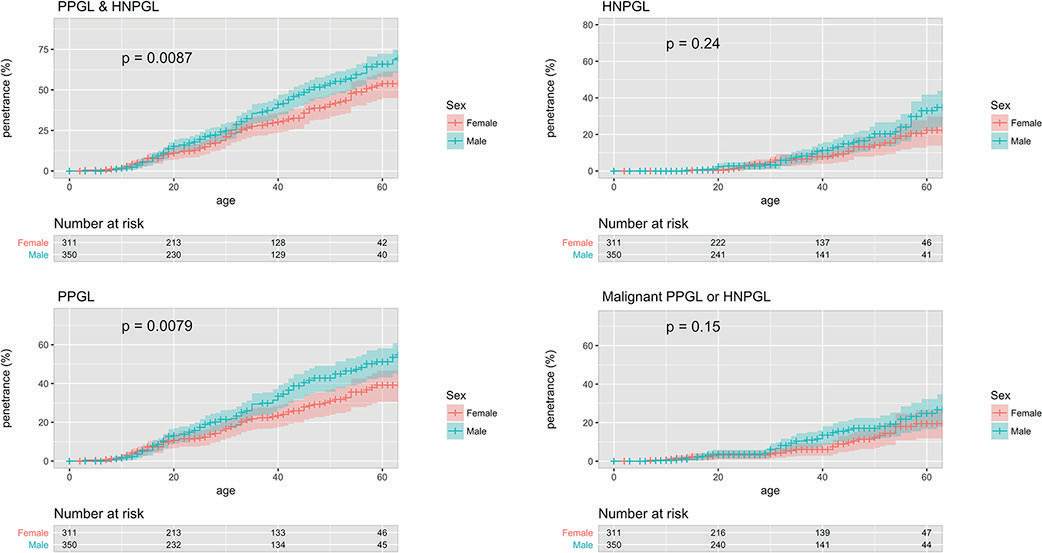
Supplementary eFigure 4

Supplementary eFigure 4

­

