# Supplementary material for: Tumour risks and genotype–phenotype correlations associated with germline variants in succinate dehydrogenase subunit genes SDHB, SDHC and SDHD
Source: J Med Genet. 2018 Jan 31;55(6):1–11. doi: 10.1136/jmedgenet-2017-105127 (PMC5992372; doi:10.1136/jmedgenet-2017-105127)
Supplement: Supplementary file 1 [file jmedgenet-2017-105127supp001.docx]

**Supplementary information - Contents**

eMethods 2

In silico protein structure analysis 2

Retrospective cohort analysis statistical methods 2

eFigures 4

References 14

# eMethods

## In silico protein structure analysis

Structure guided approaches can provide valuable insight into the molecular mechanism of mutations and their role in diseases[1–4]. mCSM and DUET are novel machine-learning methods that use graph-based structural signatures to represent the three dimensional residue environment structure in order to quantitatively predict the effects of missense mutations on protein stability, and binding affinities to their partners. To evaluate the structural effects of missense mutations, models of SDHB (NM_003000.2), based on the X-ray crystal structure of succinate dehydrogenase Ip subunit (PDB code: 2H89; sequence identity 92%), and SDHD (NM_003002.2), based on the X-ray crystal structure of small cytochrome binding protein (PDB code: 1ZP0; sequence identity 95%), were generated using Modeller[5] and MacroModel (Schrodinger, New York, NY). The DUET scoring system[6, 7] was used to predict the effects of mutations on stability, based on changes in the Gibbs free energy of folding, using the models of *SDHB* and *SDHD*. A model of the human succinate complex was built using the avian respiratory complex II (PDB code: 1ZP0) using Modeller[5]. The effects of mutations on the binding affinities between the respective subunits was predicted using mCSM-PPI.[7]

## Retrospective cohort analysis statistical methods

Standard survival analysis methods assume that mutation carriers are ascertained at random with respect

to their disease status. In the present study, the first tested individual in a family is usually someone

diagnosed with the disease. Such study designs therefore tend to lead to an over-sampling of affected

individuals, and standard analytical methods may lead to biased estimates of the risk ratios. To address this

potential bias we repeated the analysis by modelling the retrospective likelihood of the observed mutation

status conditional on the disease phenotypes.

For each disease we considered the time to disease diagnosis for the disease of interest, independently of the other disease phenotypes. We assumed a censoring process where an individual was followed from birth until the age at disease diagnosis, age at death, age at last observation, or age 85-years, whichever occurred first. In instances of no available age information (125 of the 876 *SDHB/C/D* mutation carriers), individuals were censored at age 0-years. Individuals that carried variants of unknown significance were treated as non-carriers for the purposes of these analyses.

We used retrospective cohort analysis approach to estimate the hazard ratios (HRs) separately for: (i) renal cell carcinoma (RCC); (ii) pheochromocytoma and extra-adrenal paraganglioma (PPGL); (iii) head and neck paraganglioma (HNPGL); (iv) PPGL or HNPGL; (v) tumour (RCC or PPGL or HNPGL); (vi) malignant PPGL or malignant HNPGL; and (vii) malignant tumour. We modelled the retrospective likelihood of observing genotypes conditional on the phenotypes of all individuals. These models were parameterised in terms of the log-HR and the gene mutation frequency was fixed. The *SDHB* mutation frequency was obtained from the Exome Aggregation Consortium database[8, 9]. The disease incidences were constrained over all genetic effects to agree with the assumed population disease incidences. The sex-specific RCC disease incidences were obtained from Cancer Research UK[10]. We assumed PPGL and HNPGL had an incidence of 1 per 100,000 for each age from birth. Malignant disease was assumed to account for 10% of the total disease incidence. Parameters were estimated by maximum likelihood estimation. All segregation analyses were performed using pedigree analysis software MENDEL.[11]

We tested whether assumptions regarding the gene mutation frequency and the disease incidences impacted on the results by halving and doubling the assumed values. We also investigated alternative censoring processes to ensure the results obtained by using the censoring process described above (that was designed to yield the maximum number of disease cases for each analysis) agreed with other possible approaches. Results were not sensitive to assumptions on the adopted censoring process, gene mutation frequency or disease incidences.

Absolute risks were calculated using assumed disease incidences and the estimated log-HR. The absolute risks were calculated as:

R = 1 - exp[-Λ_0_(*t*)exp{β}]

where Λ_0_(*t*) is the cumulative disease incidence up to age *t* and β is the estimated log-HR. Absolute risks calculated by this method for composite diseases (e.g. tumour being the composite of other sub-disease phenotypes) can be inflated by scenarios where the gene mutation has a relatively small influence on disease risk compared to the other disease(s), but its incidence is much larger. For example, *SDHB* has a small effect on RCC compared to the other diseases of interest, and the RCC incidence rates are much larger than the assumed incidences of PPGL or HNPGL. In this situation the large composite HR is driven by PPGL and HNPGL (not RCC), but the composite disease incidence is inflated by inclusion of the RCC incidences, hence the absolute risks for this composite disease will be inflated. Therefore, we also estimated the absolute risks using a probabilistic approach that assumes independence between diseases comprising the composite disease. This probabilistic absolute risk was calculated by:

P = 1 - Π(1-R*_i_*)

where R*_i_* is the estimated absolute risk using the previous method for disease *i*.

Association p-values were calculated from a χ^2^ 1df test. Several diseases yielded highly significant p-values (p<2.23x10^-308^), hence an approximate -log_10_(p) was calculated by:

-log_10_(p) ≈ -log*_e_*(p)/(log*_e_*(100)/2)

to describe the strength of the association. This approximation was performed using the R statistical software[12].

# eFigures

**
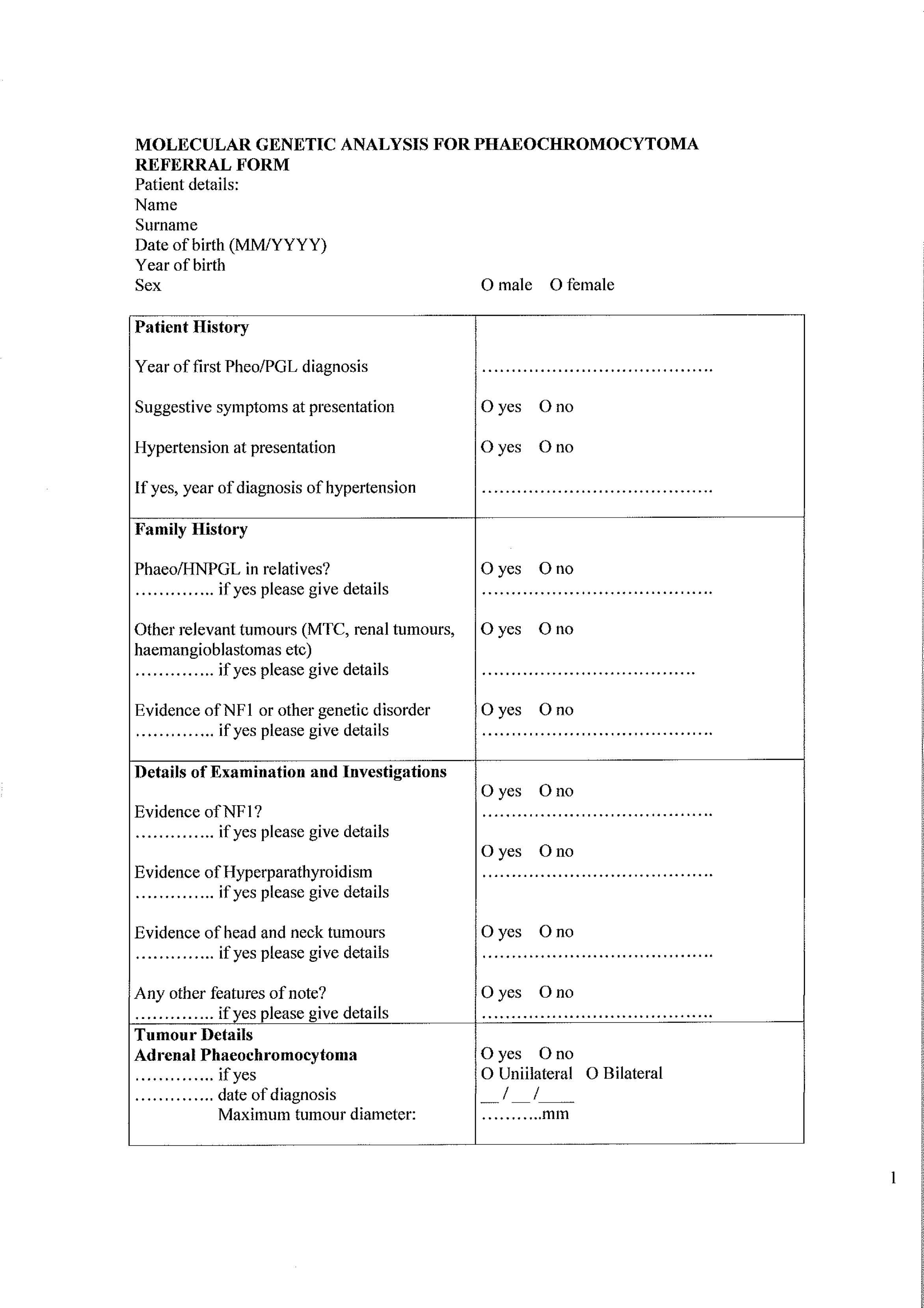

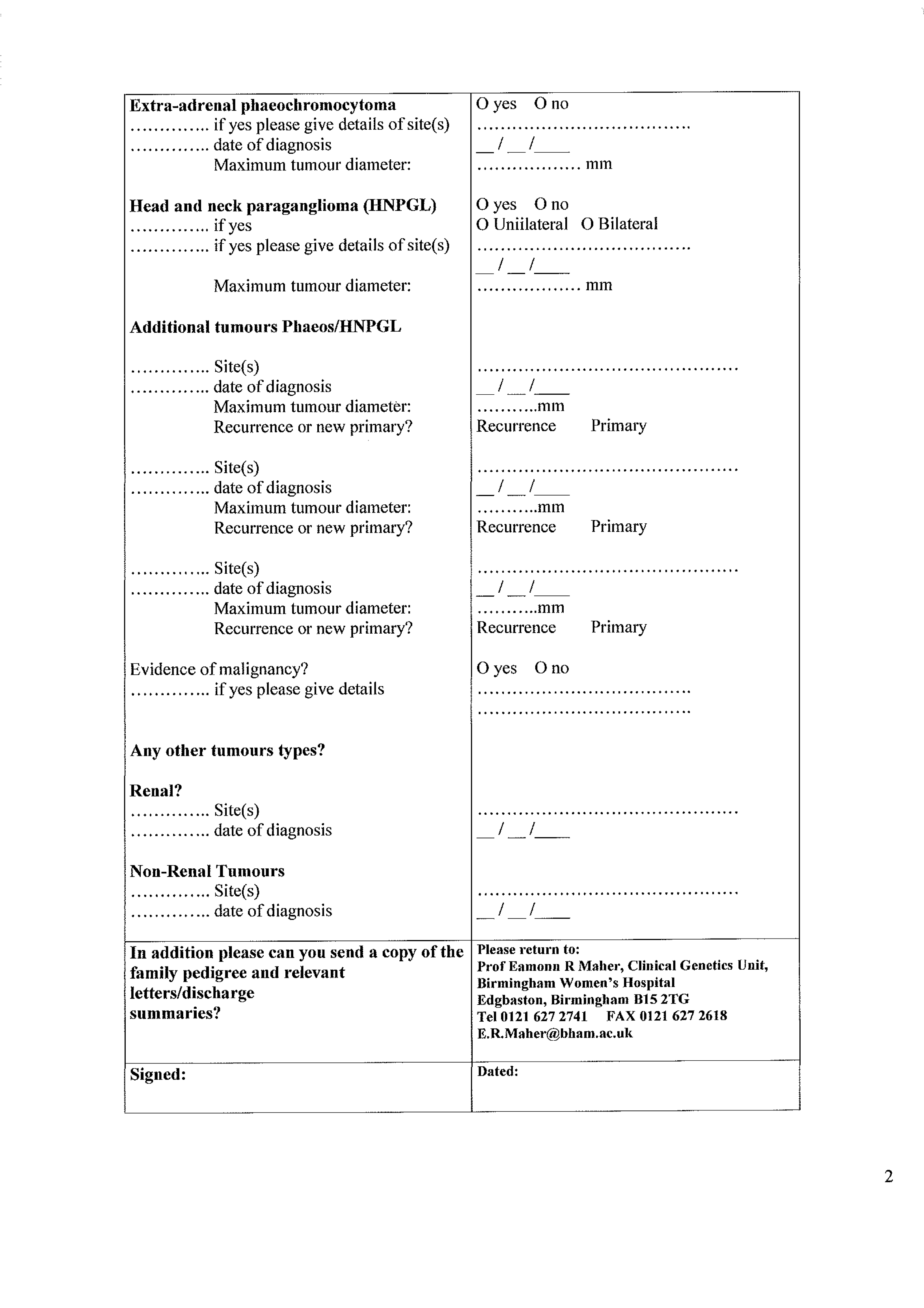
**

**eFigure 1.** Copy of the clinical information proforma sent to referring clinicians

**eFigure 2.** Of 1832 patients referred for genetic testing due to a family history of PPGL/HNPGL, numbers of probands, mutations carriers, and patients with full clinical information are shown.

**eFigure 3.** Relative frequency of the most common *SDHB*, *SDHC* and *SDHD* mutations found in probands with pheochromocytoma or paraganglioma. A large proportion of probands have one of a few common mutations.

**­**

**eFigure 4.** Difference in phenotypes of female and male *SDHB* mutation carriers, probands and non-probands, by Kaplan-Meier analysis.

PPGL – phaeochromocytoma and sympathetic paraganglioma; HNPGL – head and neck paraganglioma.

eTables

**eTable 1.** Large copy number abnormalities (CNAs) in *SDHB*, *SDHC* and *SDHD* found in this cohort. The percentage in the final column represents the proportion of probands who have the deletion or duplication in question out of all probands with large CNAs in that gene.

| **Large CNA** | **No. probands** | **Proportion large CNA probands for gene** |
| --- | --- | --- |
| SDHB Deletion exon 1 | 17 | 49% |
| SDHB Deletion exon 2 | 2 | 5.5% |
| SDHB Deletion exon 3 | 5 | 14% |
| SDHB Deletion exon 8 | 3 | 8.3% |
| SDHB Deletion exons 1-2 | 1 | 2.8% |
| SDHB Deletion exons 2-7 | 1 | 2.8% |
| SDHB Deletion exons 4-5 | 2 | 5.6% |
| SDHB Deletion whole gene | 2 | 5.6% |
| SDHB Duplication exon 1 | 1 | 2.8% |
| SDHB Duplication exons 3-5 | 1 | 2.8% |
| SDHB Duplication exons 4-5 | 1 | 2.8% |
| SDHC Deletion exon 6 | 5 | 83% |
| SDHC Deletion exons 1-3 | 1 | 17% |
| SDHD Deletion exon 4 | 3 | 100% |

**eTable 2.** Literature review of SDH mutation genotypes and penetrance of SDH-related disease, performed on in July 2017.

|  | Number SDHB mutation carriers | Number SDHB probands | % SDHB probands large CNV | Number SDHC mutation carriers | Number SDHC probands | % SDHC probands large CNV | Number SDHD mutation carriers | Number SDHD probands | % SDHD probands large CNV | Penetrance estimate - SDHB | Penetrance estimate - SDHD | Method of penetrance estimate |
| --- | --- | --- | --- | --- | --- | --- | --- | --- | --- | --- | --- | --- |
| Neumann et al. 2004[13] | 51 | 25 | - | 0 | 0 | N/A | 45 | 24 | - | 77% by age 50 | 86% by age 50 | Kalplan Meier analysis of probands and non-probands combined |
| Benn et al. 2006[14] | 82 | 43 | - | 0 | 0 | N/A | 30 | 19 | - | 45% by age 40 | 73% by age 40 | Kalplan Meier analysis of probands and non-probands combined |
| Mannelli et al. 2009[15] | 24 | 24 | 0.0 | 4 | 4 | 0.0 | 47 | 47 | 4.3 | - | - | - |
| Erlic et al. 2009[16] | 73 | 73 | 19.0 | 2 | 2 | 50.0 | 28 | 28 | 1.1 | - | - | - |
| Neumann et al. 2009[17] | 63 | 63 | 11.0 | 26 | 26 | 11.0 | 94 | 94 | 6.3 | - | - | - |
| Burnichon et al. 2009[18] | 96 | 81 | 4.9 | 16 | 14 | 14.0 | 130 | 82 | 2.4 | - | - | - |
| Solis et al. 2009[19] | 41 | 1 | N/A | 0 | 0 | N/A | 0 | 0 | N/A | 35% by age 40 |  | Kaplan Meier analysis of one large *SDHB* exon 1 deletion family |
| Schiavi et al. 2010[20] | 135 | - | - | 0 | 0 | N/A | 0 | 0 | N/A | 13% by age 50 | - | Modified segregation analysis |
| Buffett et al. 2012[21] | - | 137 | 13.0 | - | 30 | 17.0 | - | 100 | 5.0 | - | - | - |
| Hensen et al. 2012[22] | 41 | 22 | 22.7 | 2 | 2 | 50.0 | 601 | 211 | 0.5 | - | - | - |
| Rijken et al. 2016[23] | 17 | 1 | N/A | 0 | 0 | N/A | 0 | 0 | N/A | 15% by age 60 | - | Maximum likelihood estimate excluding the proband in one large *SHDB* exon 3 deletion family |
| Eijkelenkamp et al. 2016[24] | 91 | 21 | - | 0 | 0 | N/A | 0.0 | 0.0 | N/A | 12% by age 60 | - | Kaplan Meier analysis of non-probands |
| Jochmanova et al. 2017[25] | 431 | 103 | 5.8 | 0 | 0 | N/A | 0.0 | 0.0 | N/A | 20% by age 50 | - | Kaplan Meier analysis of non-probands |
| Rijken et al. 2017[26] | 195 | 65 | - | 0 | 0 | N/A | 0.0 | 0.0 | N/A | 21% by age 50 | - | Maximum likelihood estimate using all patients |
| This study | 673 | 272 | 13.2 | 43 | 26 | 23.1 | 160 | 90 | 3.3 | 22% / 24% by age 60 | 43% by age 60 | Kaplan Meier analysis of non-probands / Modified segregation analysis |

**eTable 3.** Prevalence of PPGL, HNPGL, bilateral disease and malignant disease in *SDHB*, *SDHC*, and *SDHD* mutation carriers for whom detailed clinical information is available.

|  | **SDHB** | **SDHC** | **SDHD** |
| --- | --- | --- | --- |
| **PPGL** | 191 / 584 (33%) | 4 / 33 (12%) | 23 / 134 (17%) |
| **Median age diagnosis** | 32 | 37 | 23 |
| **HNPGL** | 80 / 584 (14%) | 16 / 33 (48%) | 78 / 134 (58%) |
| **Median age diagnosis** | 43 | 42 | 40 |
| **Bilateral disease** | 22 / 584 (3.8%) | 0 / 33 (0%) | 45 / 134 (34%) |
| **Malignant disease** | 72 / 584 (12%) | 1 / 33 (3.0%) | 6 / 134 (4.5%) |

**eTable 4.** Phenotype and genotype information for all SDHB/C/D mutation carriers in this study.

Please see separate excel file.

# References

1 Gossage L, Pires DE V, Olivera-Nappa Á, Asenjo J, Bycroft M, Blundell TL, Eisen T. An integrated computational approach can classify VHL missense mutations according to risk of clear cell renal carcinoma. *Hum Mol Genet* 2014;**23**:5976–88.

2 Jafri M, Wake NC, Ascher DB, Pires DE V., Gentle D, Morris MR, Rattenberry E, Simpson MA, Trembath RC, Weber A, Woodward ER, Donaldson A, Blundell TL, Latif F, Maher ER. Germline Mutations in the CDKN2B tumor suppressor gene predispose to renal cell carcinoma. *Cancer Discov* Published Online First: 14 April 2015. doi:10.1158/2159-8290.CD-14-1096

3 Nemethova M, Radvanszky J, Kadasi L, Ascher DB, Pires DE V, Blundell TL, Porfirio B, Mannoni A, Santucci A, Milucci L, Sestini S, Biolcati G, Sorge F, Aurizi C, Aquaron R, Alsbou M, Marques Lourenço C, Ramadevi K, Ranganath LR, Gallagher JA, van Kan C, Hall AK, Olsson B, Sireau N, Ayoob H, Timmis OG, Le Quan Sang K-H, Genovese F, Imrich R, Rovensky J, Srinivasaraghavan R, Bharadwaj SK, Spiegel R, Zatkova A. Twelve novel HGD gene variants identified in 99 alkaptonuria patients: focus on ‘black bone disease’ in Italy. *Eur J Hum Genet* Published Online First: 25 March 2015. doi:10.1038/ejhg.2015.60

4 Usher JL, Ascher DB, Pires DE V, Milan AM, Blundell TL, Ranganath LR. Analysis of HGD Gene Mutations in Patients with Alkaptonuria from the United Kingdom: Identification of Novel Mutations. *JIMD Rep* Published Online First: 15 February 2015. doi:10.1007/8904_2014_380

5 Sali A, Blundell TL. Comparative protein modelling by satisfaction of spatial restraints. *J Mol Biol* 1993;**234**:779–815.

6 Pires DE V, Ascher DB, Blundell TL. DUET: a server for predicting effects of mutations on protein stability using an integrated computational approach. *Nucleic Acids Res* 2014;**42**:W314-9.

7 Pires DE V, Ascher DB, Blundell TL. mCSM: predicting the effects of mutations in proteins using graph-based signatures. *Bioinformatics* 2014;**30**:335–42.

8 Exome Aggregation Consortium (ExAC), Cambridge, MA. http://exac.broadinstitute.org

9 Lek M, Karczewski KJ, Minikel E V., Samocha KE, Banks E, Fennell T, O’Donnell-Luria AH, Ware JS, Hill AJ, Cummings BB, Tukiainen T, Birnbaum DP, Kosmicki JA, Duncan LE, Estrada K, Zhao F, Zou J, Pierce-Hoffman E, Berghout J, Cooper DN, Deflaux N, DePristo M, Do R, Flannick J, Fromer M, Gauthier L, Goldstein J, Gupta N, Howrigan D, Kiezun A, Kurki MI, Moonshine AL, Natarajan P, Orozco L, Peloso GM, Poplin R, Rivas MA, Ruano-Rubio V, Rose SA, Ruderfer DM, Shakir K, Stenson PD, Stevens C, Thomas BP, Tiao G, Tusie-Luna MT, Weisburd B, Won H-H, Yu D, Altshuler DM, Ardissino D, Boehnke M, Danesh J, Donnelly S, Elosua R, Florez JC, Gabriel SB, Getz G, Glatt SJ, Hultman CM, Kathiresan S, Laakso M, McCarroll S, McCarthy MI, McGovern D, McPherson R, Neale BM, Palotie A, Purcell SM, Saleheen D, Scharf JM, Sklar P, Sullivan PF, Tuomilehto J, Tsuang MT, Watkins HC, Wilson JG, Daly MJ, MacArthur DG, Exome Aggregation Consortium. Analysis of protein-coding genetic variation in 60,706 humans. *Nature* 2016;**536**:285–91.

10 Cancer Research UK. http://www.cancerresearchuk.org/ (accessed 4 Sep2016).

11 Lange K, Weeks D, Boehnke M, MacCluer JW, MacCluer JW. Programs for pedigree analysis: Mendel, Fisher, and dGene. *Genet Epidemiol* 1988;**5**:471–2.

12 R Core Team. R: A Language and Environment for Statistical Computing. R Foundation for Statistical Computing, Vienna, Australia. 2017.https://www.r-project.org/

13 Neumann HPH, Pawlu C, Peczkowska M, Bausch B, McWhinney SR, Muresan M, Buchta M, Franke G, Klisch J, Bley TA, Hoegerle S, Boedeker CC, Opocher G, Schipper J, Januszewicz A, Eng C. Distinct Clinical Features of Paraganglioma Syndromes Associated With SDHB and SDHD GeneMutations. *JAMA* 2004;**292**:943–51.

14 Benn DE, Gimenez-Roqueplo A-P, Reilly JR, Bertherat J, Burgess J, Byth K, Croxson M, Dahia PLM, Elston M, Gimm O, Henley D, Herman P, Murday V, Niccoli-Sire P, Pasieka JL, Rohmer V, Tucker K, Jeunemaitre X, Marsh DJ, Plouin P-F, Robinson BG. Clinical presentation and penetrance of pheochromocytoma/paraganglioma syndromes. *J Clin Endocrinol Metab* 2006;**91**:827–36.

15 Mannelli M, Castellano M, Schiavi F, Filetti S, Giacchè M, Mori L, Pignataro V, Bernini G, Giachè V, Bacca A, Biondi B, Corona G, Di Trapani G, Grossrubatscher E, Reimondo G, Arnaldi G, Giacchetti G, Veglio F, Loli P, Colao A, Ambrosio MR, Terzolo M, Letizia C, Ercolino T, Opocher G. Clinically guided genetic screening in a large cohort of italian patients with pheochromocytomas and/or functional or nonfunctional paragangliomas. *J Clin Endocrinol Metab* 2009;**94**:1541–7.

16 Erlic Z, Rybicki L, Peczkowska M, Golcher H, Kann PH, Brauckhoff M, Müssig K, Muresan M, Schäffler A, Reisch N, Schott M, Fassnacht M, Opocher G, Klose S, Fottner C, Forrer F, Plöckinger U, Petersenn S, Zabolotny D, Kollukch O, Yaremchuk S, Januszewicz A, Walz MK, Eng C, Neumann HPH. Clinical predictors and algorithm for the genetic diagnosis of pheochromocytoma patients. *Clin Cancer Res* 2009;**15**:6378–85.

17 Neumann HPH, Erlic Z, Boedeker CC, Rybicki L a, Robledo M, Hermsen M, Schiavi F, Falcioni M, Kwok P, Bauters C, Lampe K, Fischer M, Edelman E, Benn DE, Robinson BG, Wiegand S, Rasp G, Stuck B a, Hoffmann MM, Sullivan M, Sevilla M a, Weiss MM, Peczkowska M, Kubaszek A, Pigny P, Ward RL, Learoyd D, Croxson M, Zabolotny D, Yaremchuk S, Draf W, Muresan M, Lorenz RR, Knipping S, Strohm M, Dyckhoff G, Matthias C, Reisch N, Preuss SF, Esser D, Walter M a, Kaftan H, Stöver T, Fottner C, Gorgulla H, Malekpour M, Zarandy MM, Schipper J, Brase C, Glien A, Kühnemund M, Koscielny S, Schwerdtfeger P, Välimäki M, Szyfter W, Finckh U, Zerres K, Cascon A, Opocher G, Ridder GJ, Januszewicz A, Suarez C, Eng C. Clinical predictors for germline mutations in head and neck paraganglioma patients: cost reduction strategy in genetic diagnostic process as fall-out. *Cancer Res* 2009;**69**:3650–6.

18 Burnichon N, Rohmer V, Amar L, Herman P, Leboulleux S, Darrouzet V, Niccoli P, Gaillard D, Chabrier G, Chabolle F, Coupier I, Thieblot P, Lecomte P, Bertherat J, Wion-Barbot N, Murat A, Venisse A, Plouin P-F, Jeunemaitre X, Gimenez-Roqueplo A-P. The succinate dehydrogenase genetic testing in a large prospective series of patients with paragangliomas. *J Clin Endocrinol Metab* 2009;**94**:2817–27.

19 Solis DC, Burnichon N, Timmers HJLM, Raygada MJ, Kozupa a, Merino MJ, Makey D, Adams KT, Venisse a, Gimenez-Roqueplo a-P, Pacak K. Penetrance and clinical consequences of a gross SDHB deletion in a large family. *Clin Genet* 2009;**75**:354–63.

20 Schiavi F, Milne RL, Anda E, Blay P, Castellano M, Opocher G, Robledo M, Cascón A. Are we overestimating the penetrance of mutations in SDHB? *Hum Mutat* 2010;**31**:761–2.

21 Venisse A, Nau V, Roncellin I, Boccio V, Pottier N Le, Boussion M, Travers C, Simian C, Burnichon N, Abermil N, Favier J, Jeunemaitre X, Cardiovascular P. A Decade ( 2001 – 2010 ) of Genetic Testing for Pheochromocytoma and Paraganglioma. 2012;:359–66.

22 Hensen EF, van Duinen N, Jansen JC, Corssmit EPM, Tops CMJ, Romijn J a, Vriends a HJT, van der Mey a GL, Cornelisse CJ, Devilee P, Bayley JP. High prevalence of founder mutations of the succinate dehydrogenase genes in the Netherlands. *Clin Genet* 2012;**81**:284–8.

23 Rijken JA, Niemeijer ND, Corssmit EPM, Jonker MA, Leemans CR, Menko FH, Hensen EF. Low penetrance of paraganglioma and pheochromocytoma in an extended kindred with a germline SDHB exon 3 deletion. *Clin Genet* 2016;**89**:128–32.

24 Eijkelenkamp K, Osinga TE, de Jong MM, Sluiter WJ, Dullaart RPF, Links TP, Kerstens MN, van der Horst-Schrivers ANA. Calculating the optimal surveillance for head and neck paraganglioma in SDHB-mutation carriers. *Fam Cancer* Published Online First: 2016. doi:10.1007/s10689-016-9923-3

25 Jochmanova I, Wolf KI, King KS, Nambuba J, Wesley R, Martucci V, Raygada M, Adams KT, Prodanov T, Fojo AT, Lazurova I, Pacak K. SDHB-related pheochromocytoma and paraganglioma penetrance and genotype???phenotype correlations. *J Cancer Res Clin Oncol* 2017;**0**:1–15.

26 Rijken JA, Niemeijer ND, Jonker MA, Eijkelenkamp K, Jansen JC, van Berkel A, M Timmers HJL, Kunst HPM, Bisschop PHLT, Kerstens MN, Dreijerink KMA, van Dooren MF, van der Horst-Schrivers ANA, Hes FJ, René Leemans C, Corssmit EPM, Hensen EF. The Penetrance of Paraganglioma and Pheochromocytoma in *SDHB* germline mutation carriers. *Clin Genet* Published Online First: 14 May 2017. doi:10.1111/cge.13055
